# Aerobic Training Is Better Than Resistance Training on Cardiac Function and Autonomic Modulation in Female ob/ob Mice

**DOI:** 10.3389/fphys.2019.01464

**Published:** 2019-12-05

**Authors:** Filipe Fernandes Stoyell-Conti, Maria-Claudia Irigoyen, Michelle Sartori, Amanda Aparecida Ribeiro, Fernando dos Santos, Jacqueline Freire Machi, Diego Mendrot Taboas Figueroa, Bruno Rodrigues, Kátia De Angelis

**Affiliations:** ^1^Health Professional Division, College of Pharmacy, Nova Southeastern University, Fort Lauderdale, FL, United States; ^2^Translational Physiology Laboratory, Universidade Nove de Julho (UNINOVE), São Paulo, Brazil; ^3^Hypertension Unit, Medical School, Heart Institute (InCor), University of São Paulo, São Paulo, Brazil; ^4^Department of Molecular and Cellular Pharmacology, University of Miami (UM), Coral Gables, FL, United States; ^5^Department of Adapted Physical Activity, Faculty of Physical Education, Universidade Estadual de Campinas, Campinas, Brazil; ^6^Department of Physiology, Federal University of São Paulo (UNIFESP), São Paulo, Brazil

**Keywords:** obesity, diastolic dysfunction, autonomic nervous system, exercise training, female

## Abstract

**Objective:** This study evaluated the effects of aerobic, resistance, and combined exercise training on cardiac function and autonomic modulation in female ob/ob mice.

**Methods:** Four-week-old female wild type and obese (ob/ob) mice were divided into five groups (*n* = 8): control (WT), obese (OB) obese + aerobic training (OBA), obese + resistance training (OBR), and obese + combined training (OBC). The exercise training was performed on treadmill and/or ladder at 40–60% maximum test during 8 weeks. Cardiac function was measured using echo machine. Heart rate variability (HRV) was evaluated in the time and frequency domain.

**Results:** OB group presented higher body weight gain (~600%), glycemia (~44%) and glucose intolerance (~150%), reduction of cardiac vagal modulation, evidenced by a lower RMMSD (~56%), total power and high frequency band, and a higher isovolumic relaxation time (IVRT) (~24%) in relation to the WT group. Aerobic and combined training led to a lower IVRT (OBA: ~14%; OBC: ~14%) and myocardial global index (OBA: ~37%; OBC: ~44%). The OBA group presented an increased in vagal indexes of HRV than the other ob/ob groups. A negative correlation was observed between the delta of aerobic exercise capacity and MPI (*r* = 0.45; *p* = 0.002) and exercise capacity and body weight gain (*r* = 0.39; *p* = 0.002).

**Conclusion:** Only the obese females underwent to aerobic exercise training showed improvement in cardiac function and HRV. Moreover, the aerobic exercise capacity as well as a greater responsivity to aerobic exercise training is intimately associated with these improvements, reinforcing the importance of aerobic exercise training to this population.

## Introduction

Obesity is an important risk factor for cardiovascular disease (CVD), such as left ventricular dysfunction, congestive heart failure, stroke, and cardiac arrhythmias. It is related to several cardiovascular risk factors as well as dyslipidemia, hypertension, inflammation, insulin resistance, and type 2 diabetes mellitus (Klein, 2004). Indeed, more than 80% of diabetic subject present overweight or are obese (Centers for Disease Control and Prevention (CDC), 2004). Diabetes *per se* has long been recognized to be an independent risk factor for CVD and it is a particularly strong risk factor among women and among the growing elderly population (A Joint Editorial Statement by the American Diabetes Association; the National Heart, Lung, and Blood Institute; the Juvenile Diabetes Foundation International; the National Institute of Diabetes and Digestive and Kidney Diseases; and the American Heart Association, 1999). The cardiovascular complications of diabetes mellitus include coronary heart disease (CHD), stroke, peripheral arterial disease, nephropathy, retinopathy, and possibly cardiomyopathy and neuropathy (Grundy et al., 1999). Recently, we showed that female adult ob/ob mice, a model of obesity and type 2 diabetes, present discrete diastolic dysfunction accompanied by autonomic disorder, which is associated with inflammation and oxidative stress (Sartori et al., 2017).

Among the factors that contribute to the obesity and diabetes development, the physical inactivity is highly prevalent. Epidemiological evidence supports the use of increased exercise in preventing age-associated weight and fat gains (National Institutes of Health, 1998). Weight loss in obese patients can improve or prevent many of the obesity-related cardiovascular risk factors (Klein, 2004). Despite the majority of the studies have verified the effects of aerobic exercise training (e.g., running or jogging, swimming, cycling, and walking) in diabetic patients, the number of studies about the effects of resistance exercise training (e.g., weightlifting) in this population and more recently about the effects of combined exercise training (aerobic associated with resistance training) has increased. These studies show a positive effect of combined training on body composition (BMI, fat mass, waist or waist to hip), a decrease of cardiovascular risk, an improvement of strength, muscle endurance, and exercise capacity, and an increase or status quo of insulin sensitivity, glucose tolerance, glycaemia, and HbA1c concentration (Albright et al., 2000; Balducci et al., 2004; Wagner et al., 2006).

It is important to note that, in this study, the term “combined exercise training” differs from “concurrent exercise training,” which investigates the fact that aerobic training performed immediately before or after strength training in the same training session limits muscle strength, power, and hypertrophy gains (Docherty and Sporer, 2000; Mikkola et al., 2012). Here, “combined exercise training” means that the animals performed both aerobic and resistance training, instead only one type of exercise.

However, there are few studies which verified the effects of three different protocols of exercise training in an obesity and diabetic condition, especially in females, which may have different adaptations from males due the female hormones and have expressive and increasing prevalence of morbimortality related to cardiometabolic diseases (Colditz et al., 1995; Barnes, 2011). Thus, the aim of this study was to verify the effects of aerobic, resistance, and combined exercise training on cardiac function and autonomic modulation in female wild-type and ob/ob mice, a model of obesity and diabetes.

## Methods

Four-week-old female wild-type and ob/ob mice were obtained from the Animals Facilities of the Federal University of Sao Paulo. The ob/ob mice lack functional leptin. They are grossly overweight and hyperphagic, particularly at young ages, and develop severe insulin resistance. They have been used as a model for obesity and as a rich source of pancreatic islets with high insulin release capacity (Lindström, 2007). The mice were divided into wild-type sedentary group (WT; *n* = 8), ob/ob sedentary group (OB; *n* = 8), ob/ob + aerobic exercise training group (OBA; *n* = 8), ob/ob + resistance exercise training group (OBR; *n* = 8), and ob/ob combined exercise training group (OBC; *n* = 8). All surgical procedures and protocols were approved by the ethics committee of the University of Sao Paulo and were conducted in accordance with the Guide for the Care and Use of Laboratory Animals, issued by the National Institutes of Health. All surgeries were performed under anesthesia, and all efforts were made to minimize suffering. All *in vivo* evaluations and euthanasia were conducted on not ovulatory phase of estrous cycle.

### Aerobic Exercise Training Protocol

All animals were adapted to the treadmill (10 min/day; 0.3 km/h) for 5 days before beginning the exercise training protocol and underwent a maximal treadmill test, as described previously (Rodrigues et al., 2007). The tests were performed at the beginning (1st week of exercise training protocol), at the middle (4th week of exercise training protocol), and at the end of protocol (8th week of exercise training protocol). The purpose of these tests was to determine exercise capacity and to prescribe exercise training intensity.

**Figure 1:**
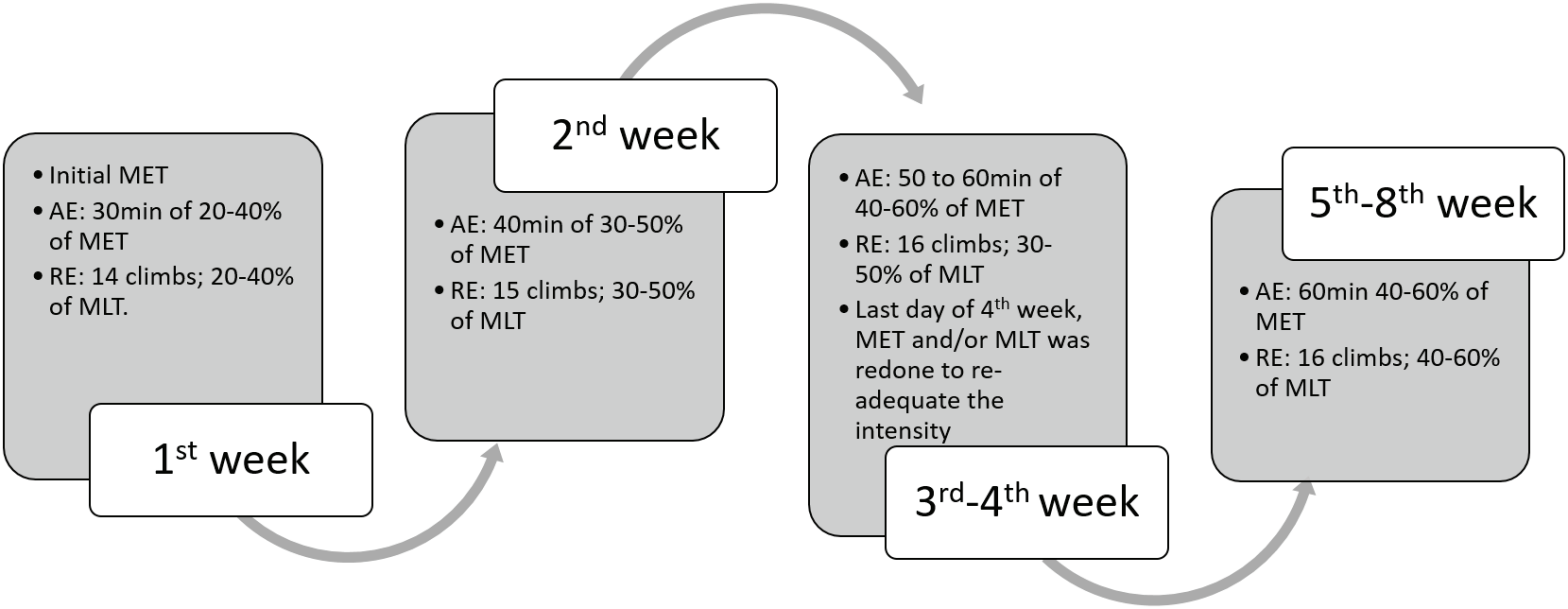
Schematic figure of aerobic and resistance exercise session. Aerobic exercise training was performed 5 days/week, during 8 weeks. Resistance exercise training was performed 5 days/week, during 8 weeks. Combined exercise training was performed 5 days/week, in alternate days (1 day aerobic and the other day resistance training) during 8 weeks. AE, aerobic exercise; RT, resistance exercise; MET, maximal exercise test; MLT, maximal load test.

Exercise training was performed on a motorized treadmill (Imbramed TK-01) at low-moderate intensity (40–60% maximal running speed) for 1 h a day, 5 days/week for 8 weeks, with a gradual increase in speed from 0.3 to 1.2 km/h (Figure 1) (Irigoyen et al., 2005).

### Resistance Exercise Training Protocol

This protocol was performed in a ladder for mice, as previously described in detail in Sanches et al. (2014). The animals were adapted to the act of climbing for five consecutive days, before the maximal load test. The test consisted of an initial load of 75% of the body weight. After completing the first climb, a 2-min resting period preceded the following climb. For this next climb, the load was increased by another 15, 25 or 40% of body weight in the test performed at 1st, 4th, and 8th weeks of the protocol, respectively. This increment was repeated successively until the animal could not complete the climb bearing the load (maximum of six climbs). The maximal tests were performed at the beginning (1st week of exercise training protocol), at the middle (4th week of exercise training protocol), and at the end of protocol (8th week of exercise training protocol). The resistance exercise training protocol was performed during 8 weeks, 5 days a week, and at moderate intensity (40–60% of the maximal load) with 15 climbs per session and a 1-min time interval between climbs (Figure 1).

### Combined Exercise Training

Combined exercise training was performed on a motor treadmill (aerobic training) and in a ladder adapted to rats (resistance training), in alternate days, at low-moderate intensity (40–60% maximal running speed and maximal load test), 5 days/week, during 8 week (Figure 1) (Conti et al., 2015).

### Metabolic Evaluations

Blood glucose and triglyceride concentrations were measured (Accucheck and Accutrend, Roche), after 4-h fasting. Glucose tolerance test (GTT) was performed at 8th week (last week) of the experimental protocol. Mice were fasted, with animals receiving only water, for 6 h. Blood samples were taken from a tail cut at 0, 15, 30, 60, and 90 min after i.p. glucose load (1.5 g/kg). Blood glucose was determined by Accu-Chek Advantage Blood Glucose Monitor (Roche Diagnostic Corporation, Indianapolis, IN).

### Echocardiographic Evaluation

Echocardiography was performed by an observer blinded to the groups, according to the guidelines of the American Society of Echocardiography. Mice were anesthetized with ketamine (50 mg/kg) and xylazine (10 mg/kg), and images were obtained using a Sequoia 512 ultrasound system (ACUSON, Mountain View, CA, USA) with a 10–14 MHz linear transducer for the measurement systolic function [ejection fraction (EF) and fractional shortening (FS)] and diastolic function [left ventricle intern diameter in diastole (LVIDd), left ventricular isovolumetric relaxation time (IVRT), and E wave/A wave ratio (E/A)] and global cardiac function [myocardial performance index (MPI)]. Left ventricle (LV) mass was calculated by using the following formula, assuming a spherical LV geometry and validated in rats: LV mass = 1,047 × [(LVd + PWd + IVSd)3 −LVd3], where 1,047 is the specific gravity of muscle (Wichi et al., 2007).

### Hemodynamic Measurements

After 8 weeks, mice were anesthetized (ketamine-xylazine 80:40 mg/kg i.p.) and polyethylene-tipped Tygon cannulas (4 cm of PE-08 connected to 2 cm of PE-50, Clay Adams) filled with heparinized saline were inserted into the carotid artery and jugular vein for direct measurements of arterial pressure and drug administration, respectively. The free ends of the cannulas were tunneled subcutaneously and exteriorized at the top of the skull. Two days after the catheter placement, hemodynamic measurements were made in conscious, freely moving mice. The arterial cannula was connected to a transducer (Blood Pressure XDCR, Kent© Scientific), and blood pressure signals were recorded for a 20-min period using a microcomputer equipped with an analog-to-digital converter (CODAS, 4-kHz sampling frequency, Dataq Instruments). The recorded data were analyzed on a beat-to-beat basis to quantify changes in blood pressure and heart rate (HR) (Wichi et al., 2007; Heeren et al., 2009).

### Autonomic Modulation

The pulse interval (PI) signals for HRV were captured as described above. Before any calculation of HRV, the recordings were edited and corrected manually for ectopic beats, arrhythmias, noise, and trends using the Windaq software. Then it was followed by PI detection by using Windaq software and the detected PI was edited manually to ensure all PI were marked. The file was saved as lotus file, which was readable by MS Excel. This Lotus file was opened in Excel and cumulative values of PIs were converted into individual PI series. Thus, the intervals between successive pulse-to-pulse interval or instantaneous heart rate values for each cardiac cycle were determined.

*Linear analyses*: Time-domain variables were: root mean square of the successive differences (RMSSD) and standard deviation of pulse interval (SD-PI). Power spectral density was obtained by the fast Fourier transformation. Spectral power for low- (LF: 0.1–1.0 Hz), and high- (HF: 1.0–5.0 Hz) frequency bands was calculated by means of power spectrum density integration within each frequency bandwidth, using a customized routine (MATLAB 6.0, Mathworks) (Wichi et al., 2007; Heeren et al., 2009).

*Non-linear analyses: Poincaré Plot Analysis* – The Poincaré plot is a graph in which each PI interval (intervals between successive heart beats) is plotted as a function of the previous PI interval. Briefly, scattergrams of successive PI intervals were plotted for the entire period, and the standard deviation (SD) of instantaneous PI interval variability and the SD of continuous variability (SD2) were then analyzed. The SD1 is an index of the instantaneous recording of the variability of beat-to-beat and represents the parasympathetic activity, whereas the SD2 index represents the long-term HRV and reflects the overall variability (Raimundo et al., 2013).

*Detrended Fluctuation Analysis*: The detrended fluctuation analysis technique was used to quantify the fractal scaling properties of short- and intermediate-term PI interval time series. The root-mean-square fluctuation of integrated and detrended time series is measured at different observation windows and plotted against the size of the observation window on a log-log scale. The details of this method have been described elsewhere (Lombardi et al., 1996).

*Approximate Entropy and Sample Entropy*: Approximate entropy (ApEn) was proposed by Pincus in 1991 as a method for measuring regularity and complexity in time series and has been successfully used to analyze physiological time series (Pincus, 1991). As a quantification of regularity in data, ApEn can be used to measure the abnormality and complexity of signals. The more complex and irregular the signal is, the higher the ApEn value. The sample entropy (SampEn) was used to assess the complexity of the HR “signal” under the different conditions. SampEn measures the likelihood that runs of patterns that are close to each other will remain close in the next incremental comparisons (Pincus, 1991).

### Statistical Analysis

Data are expressed as mean ± SE and median ± interquartile range. The normality of data was tested by Kolmogorov-Smirnov. One-way ANOVA or Dunn’s tests were used to compare the groups. Pearson correlation was used to analyze the association between variables. The significance level was established at *p* ≤ 0.05.

## Results

**Figure 2:**
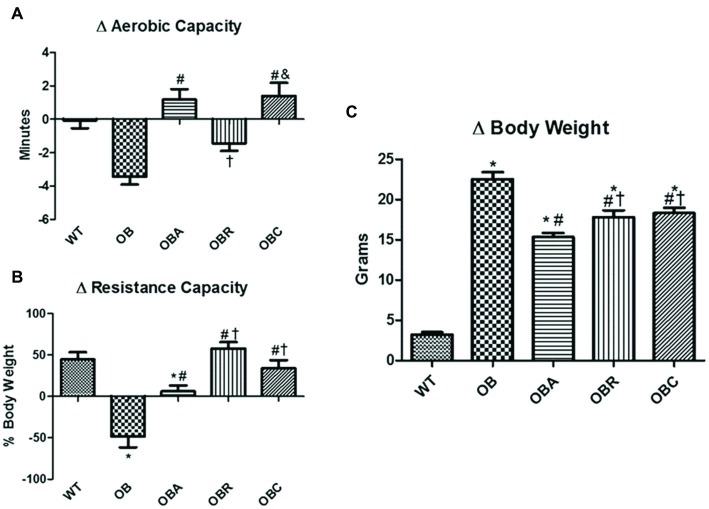
**(A)** Delta of aerobic capacity; **(B)** delta of resistance capacity, and **(C)** delta of body weight. Values are mean ± SE. ^*^p < 0.05 vs. WT; ^#^*p* < 0.05 vs. OB; ^†^*p* < 0.05 vs. OBA; ^&^*p* < 0.05 vs. OBR.

As shown in Figure 2, the ob/ob groups presented higher body weight gain compared to the wild type group (OB: 22.5 ± 0.8 vs. WT: 3.2 ± 0.2 g). On the other hand, all three groups that underwent exercise training protocol showed a lower body weight gain than the sedentary group, being the aerobic exercise training a better approach when compared to the resistance and combined exercise training (OBA: 15.3 ± 0.5; OBR: 17.8 ± 0.8; OBC: 18.3 ± 0.6 g). The glycaemia (OB: 174 ± 9 vs. WT: 121 ± 3 mg/dl), as well as the glucose tolerance (OB: 38,491 ± 3,351 vs. WT: 15,424 ± 1,272 mg/dl/%) were higher in the ob/ob animals in relation to wild type group. Once more, all three groups that underwent exercise training protocol presented a reduction of both glycaemia (OBA: 139 ± 6; OBR: 145 ± 7; OBC: 143 ± 6 mg/dl) and glucose tolerance (OBA: 31,995 ± 2,084; OBR: 27,908 ± 1,489; OBC: 26,319 ± 938 mg/dl/%) in relation to the OB group.

To verify whether the ob/ob animals present a decreased exercise capacity, we performed a maximal exercise test, either for aerobic and resistance capacity. Indeed, the OB group presented a reduction of both aerobic (OB: −3.4 ± 0.4 min) and resistance (OB: −48.4 ± 13.0 g) capacity. It was observed a specificity of training, those animals that underwent aerobic exercise training (OBA and OBC groups) showed an increase in aerobic capacity (OBA: 1.2 ± 0.6; OBR: −1.4 ± 0.4; OBC: 1.4 ± 0.8 min), and those animals that underwent resistance exercise training (OBR and OBC) showed an increase in resistance capacity (OBA: 6.3 ± 7.0; OBR: 57.8 ± 7.7; OBC: 34.0 ± 9.6 g) along the protocol (Figure 2).

**Figure 3:**
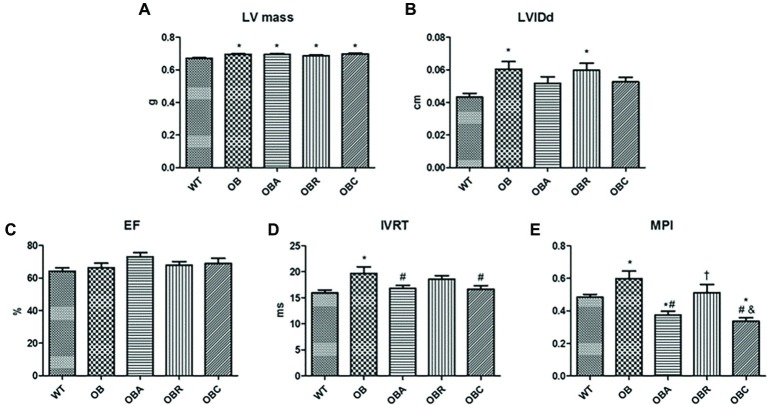
**(A)** Left ventricle mass; **(B)** left ventricle intern diameter in diastole; **(C)** ejection fraction; **(D)** isovolumic relaxation time; **(E)** myocardial performance index. Values are mean ± SE. ^*^p < 0.05 vs. WT; ^#^*p* < 0.05 vs. OB; ^†^*p* < 0.05 vs. OBA; ^&^*p* < 0.05 vs. OBR.

The obese groups presented a higher LV mass in relation to the control group (OB: 0.695 ± 0.004; OBA: 0.694 ± 0.006; OBR: 0.686 ± 0.006; OBC: 0.697 ± 0.004 vs. WT: 0.671 ± 0.004 g). The OB and OBR groups showed an increased LVIDd when compared to the control group (WT: 0.043 ± 0.002; OB: 0.060 ± 0.004; OBA: 0.051 ± 0.003; OBR: 0.059 ± 0.004; OBC: 0.052 ± 0.002 mm). There was no difference in EF among groups (WT: 64 ± 2; OB: 66 ± 2; OBA: 73 ± 2; OBR: 67 ± 2; OBC: 69 ± 3%). The IVRT was increased in the OB group in relation to the control group, and only the animals underwent aerobic exercise training showed a reduction of this parameter (WT: 15.9 ± 0.5; OB: 19.7 ± 1.2; OBA: 16.8 ± 0.6; OBR: 18.5 ± 0.6; OBC: 16.6 ± 0.7 ms). The OB group presented a higher MPI than control group, and once again, only the aerobic exercise training was able to normalize it (WT: 0.48 ± 0.01; OB: 0.59 ± 0.04; OBA: 0.37 ± 0.02; OBR: 0.51 ± 0.05; OBC: 0.33 ± 0.02) (Figure 3).

**Figure 4:**
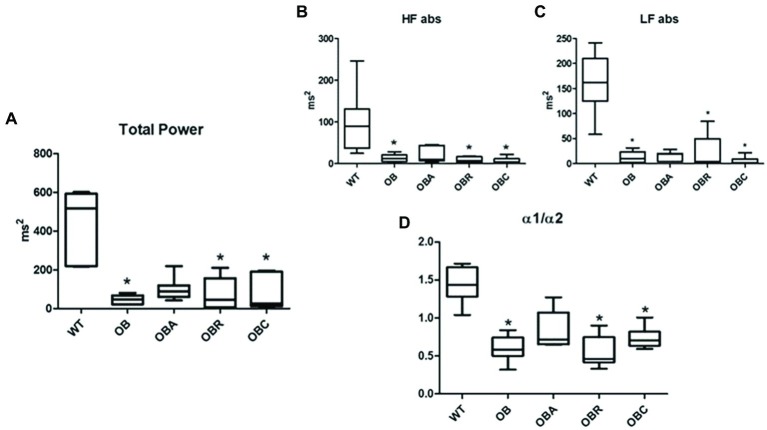
**(A)** Total power; **(B)** high frequency (absolute values); **(C)** low frequency (absolute values); **(D)** ratio alpha 1/alpha 2. Values are median ± interquartile range. ^*^p < 0.05 vs. WT.

It was observed a positive correlation between EF and aerobic exercise capacity (*r* = 0.31; *p* = 0.03) and negative correlations between the delta of aerobic exercise capacity and the MPI (*r* = 0.45; *p* = 0.002) and between the exercise capacity and the body weight gain (*r* = 0.39; *p* = 0.002) (Figure 4).

**Figure 5:**
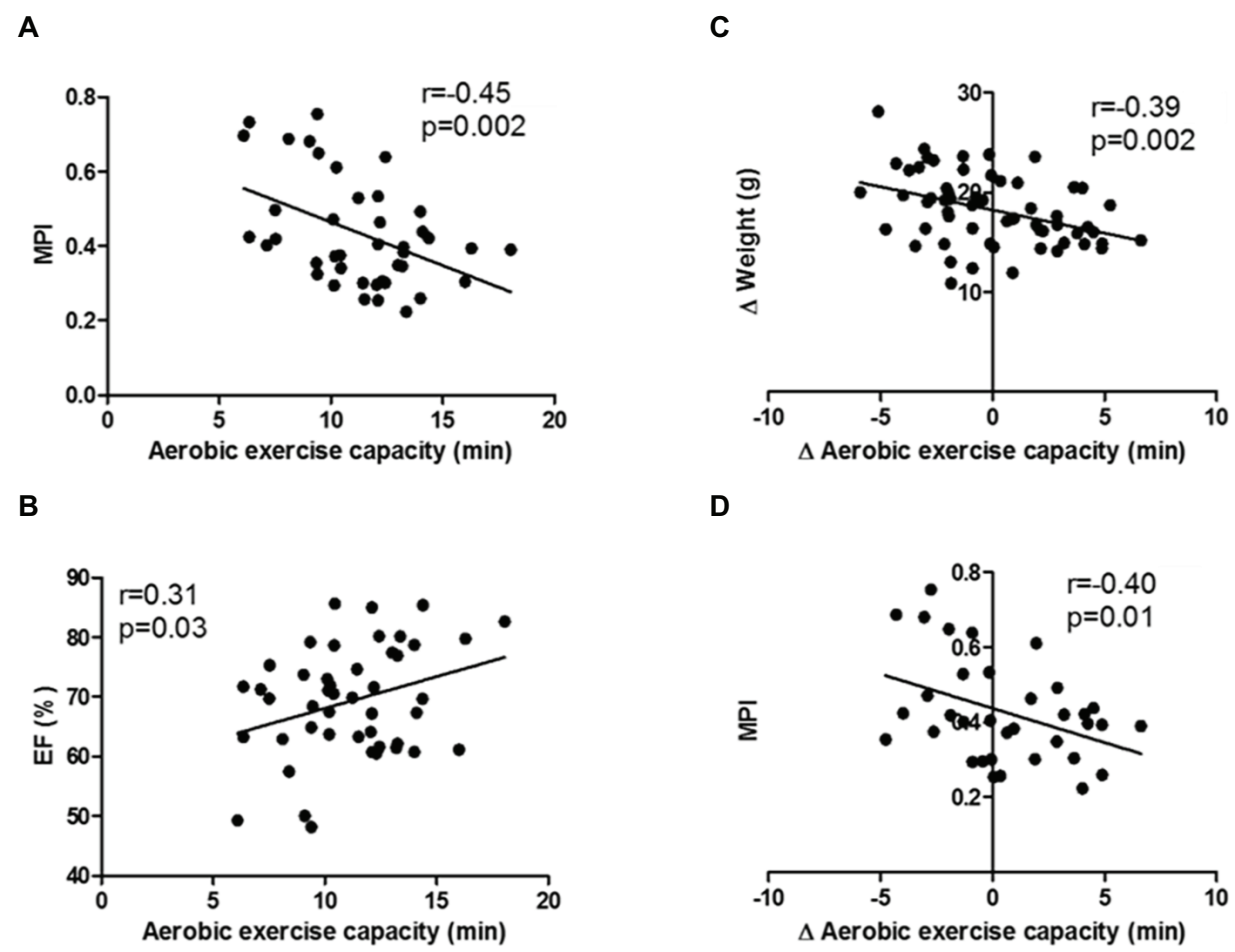
Correlation between **(A)** MPI and aerobic exercise capacity; **(B)** ejection fraction and aerobic exercise capacity; **(C)** delta of weight and delta of aerobic exercise capacity; **(D)** MPI and delta of aerobic exercise capacity.

The ob/ob animals presented lower RMSSD index, SD-PI, SD1, SD2, and alpha 1 than control group. Alpha 2 was higher in the OB group when compared to the control group. It was observed that HFabs, LFabs, Total Power and alpha1/alpha2 were reduced in the OB, OBT and OBC groups when compared to the C group. On the other hand, the animals underwent aerobic exercise training prevented the decrease in alpha 2, and the increase in HFabs, LFabs, total power, and alpha1/alpha2 parameters (Table 1; Figure 5).

**Table 1:** Linear and non-linear analyses of autonomic modulation in all groups studies.

	W	OB	OBA	OBR	OBC
**Linear analyses**					
RMSSD (ms)	19.7 ± 3.5	8.5 ± 1.8^*^	8.4 ± 1.2^*^	8.3 ± 2.6^*^	5.6 ± 0.7^*^
SD-PI (ms)	20.0 ± 1.6	11.5 ± 1.8^*^	9.8 ± 1.5^*^	14.9 ± 3.9^*^	7.4 ± 1.9^*^
**Non-linear analyses**					
SD1 (ms)	13.9 ± 2.5	4.5 ± 0.5^*^	5.9 ± 0.8^*^	4.9 ± 0.7^*^	4.2 ± 0.4^*^
SD2 (ms)	24.2 ± 1.5	13.0 ± 1.5^*^	12.7 ± 0.8^*^	10.4 ± 3.4^*^	10.6 ± 3.1^*^
Alpha 1	1.00 ± 0.09	0.55 ± 0.05^*^	0.67 ± 0.06^*^	0.43 ± 0.07^*^	0.50 ± 0.04^*^
Alpha 2	0.61 ± 0.04	0.97 ± 0.03^*^	0.78 ± 0.05^#^	0.83 ± 0.04	0.70 ± 0.08^#^
SampEn	1.56 ± 0.09	1.72 ± 0.03	1.58 ± 0.10	1.49 ± 0.16	1.65 ± 0.08
ApEn	1.41 ± 0.05	1.47 ± 0.02	1.41 ± 0.06	1.39 ± 0.07	1.46 ± 0.04

* p < 0.05 vs. WT;

# p < 0.05 vs. OB;

*Values are mean ± SE*.

*RMSSD, root mean square of the successive differences; SD-PI, standard deviation of pulse interval; Poincare plot (SD1 e SD2); detrended fluctuation analysis (Alpha 1 and Alpha 2); Sample entropy (SampEn), and approximate entropy (ApEn)*.

## Discussion

In the USA, 32% of white and 53% of black women are obese according to The Centers for Disease Control and Prevention report. Obesity is the leading risk factor for type 2 diabetes. Both obesity and diabetes mellitus are important independent risk factors for the development of cardiovascular disease. And, indeed, cardiovascular disease remains the leading cause of death in women (Colditz et al., 1995; Barnes, 2011).

The ob/ob mice are known as an optimal model of obesity and type 2 diabetes. Due its lack of functional leptin, the ob/ob mice present hyperphagia, which contributes to obesity, insulin resistance, and type 2 diabetes development (Friedman et al., 1991). Thus, the aim of this study was to evaluate the effects of three different types of exercise training (aerobic, resisted, and combined) on autonomic modulation and cardiac function in female ob/ob mice, an experimental model of obesity and type 2 diabetes. Despite the positive effects of all three types of exercise training in attenuating weight gain, as well as reducing glycaemia and glucose intolerance, the two major and new findings of this study were: (1) only the animals underwent to aerobic exercise training showed an improvement in cardiac function (OBA and OBC groups) and HRV (OBA group); (2) the aerobic exercise capacity as well as a greater responsivity to aerobic exercise training are intimately associated with those improvements.

All three exercise training protocols promoted a lower gain weight compared to the obese sedentary group. However, the aerobic exercise training was more effective. Recently, Evangelista et al. (2015) demonstrated, in ob/ob mice, that the aerobic exercise promoted lower gain weight along the protocol, as well as led to a greater carbohydrate and lipids oxidation in the exercise group in relation to the sedentary group. Indeed, several studies indicate that the aerobic exercise training is a better approach for losing weight than the resistance exercise training. Willis et al. (2012) compared the effects of aerobic, resistance, and combined exercise training on body mass and fat mass in overweight or obese adults. They observed that the aerobic exercise training decreases both body weight and fat mass significantly more than does resistance exercise training. While the two modes of exercise produced statistically similar changes in body fat percentage, these changes were driven by different mechanisms, where resistance exercise training increased lean body mass and aerobic exercise training decreased fat mass. The combined exercise training did not result in a greater loss of fat mass or body mass over aerobic, corroborating with the data of the present study. Although, the aerobic exercise training resulted in a lower weight gain, all three types of exercise training were able to reduce this parameter. This finding is important as it shows that weight loss is associated with improved diabetes control and CVD risk factors and reduced medicine use in patients with type 2 diabetes (Look AHEAD Research Group et al., 2007).

Besides an altered metabolic profile, a variety of adaptations/alterations in cardiac structure and function occur in the individual with obesity as adipose tissue accumulates in excess amounts, even in the absence of comorbidities (Poirier, 2006). The obese animals showed an increased LV mass when compared to the control group. Lauer et al. (1991) analyzed 3,922 participants of the Framingham Heart Study, and they showed that obesity is significantly correlated with left ventricular mass, even after controlling for age and blood pressure. The increase in left ventricular mass associated with increasing adiposity reflects increases in both left ventricular wall thickness and left ventricular internal dimension. Previously, we showed that ob/ob mice present diastolic dysfunction when compared to the wild type animals (Sartori et al., 2017). Indeed, in the present study, ob/ob animals presented an impairment on LVIDd, IVRT, and MPI, characterizing a diastolic dysfunction. Longer durations of obesity are associated with poorer left ventricular systolic function and greater impairment of left ventricular diastolic function (Alpert et al., 1995). Because of the presence of nonspecific symptoms, the evaluation of the presence of left ventricular diastolic dysfunction is clinically important in obese subjects (Chakko et al., 1991).

It is important to emphasize that an impairment of diastolic function could result in the inability to increase cardiac output adequately and can limit exercise capacity. The sedentary ob/ob animals showed a lower aerobic exercise capacity. On the other hand, only the animals that underwent exercise training protocol with an aerobic component (OBA and OBC groups) showed a greater aerobic exercise capacity as well as an improvement on LVIDd, IVRT, and MPI. Animal studies have shown increased rates of myocardial relaxation and improved diastolic function after aerobic exercise training (Wisløff et al., 2001; Bito et al., 2010), possibly mediated by enhanced calcium uptake by the sarcoplasmic reticulum (Tate et al., 1990) or more efficient excitation-contraction coupling (Gwathmey et al., 1990). Interestingly, even not showing a significantly difference among groups for ejection fraction, it was observed a positive correlation between EF and aerobic exercise capacity. In addition, it was observed a negative correlation between MPI and aerobic exercise capacity. The MPI, also known as the RIMP or Tei index, is a global estimate of both systolic and diastolic function of the right ventricle. It is based on the relationship between ejection and non-ejection work of the heart, and lower values represent a better prognostic (Tei, 1995). Thus, a greater aerobic exercise capacity is associated to a better cardiac function. Therefore, these results suggest that aerobic exercise training is a better approach than resistance exercise training to the management of obesity induced-cardiac dysfunction in females.

Moreover, a negative correlation was observed between the delta of aerobic exercise capacity and MPI and body weight gain. This finding suggests that those animals are more responsive to aerobic exercise training, presenting better response to the same stimulus, also they are more responsive to aerobic exercise training effects, showing lower weight gain and improvement on cardiac function. Regarding the training volume, if we consider the time × intensity × frequency of training protocols applied in the present study, all animals trained ~1 h per/day, 5 days/week, at 40–60% of maximal exercise test, suggest similar volume. However, it is important to remember that aerobic and resistance training present different characteristics of metabolism and prescription; which, for example, allow the animals under an aerobic exercise to run almost continuously for 1 h and the animals under resistance exercise training have to have a recovery period between the exercise series. Nevertheless, these characteristics do not allow a perfect comparison of “volume” between these two types of training, and they are the same or so close to what is observed in clinical practice, reinforcing the translational approach of the present study.

Certainly, aerobic exercise capacity plays a pivotal role in cardiac function and metabolic profile. Numerous studies have demonstrated that low exercise capacity is a stronger predictor of morbidity and mortality relative to other frequently reported risk factors including hypertension, type II diabetes, obesity, and smoking (Myers et al., 2002; Kavanagh et al., 2003; Kokkinos et al., 2008). Wisløff et al. (2005) showed, *via* two-way artificial selective breeding of rats for low (LCR) and high (HCR) intrinsic endurance exercise capacity, that adult LCR rats develop cardiovascular risks consistent with the metabolic syndrome including large gain in visceral adiposity, increased blood pressure, dyslipidemia, endothelial dysfunction occurring within carotid arteries, and insulin resistance.

It is important to remind that autonomic nervous system is an imperative contributor to the regulation of both the cardiovascular system and energy expenditure, and it is assumed to play a pivotal role in the pathophysiology of obesity and diabetes and related complications. Diabetic patient, in some cases, presents cardiac autonomic neuropathy (CAN), which is caused by damage to the autonomic nerve fibers that innervate the heart and blood vessels and leads to abnormalities in cardiovascular dynamics (Vinik and Erbas, 2013) and it may be diagnostic by a decrease in heart rate variability (HRV) (Schönauer et al., 2008). Considering that the HRV analysis represents an effective method for evaluating the function of the autonomic nervous system, we decided to perform the HRV analysis to verify if this experimental model of diabetes and obesity presents similar autonomic dysfunction as seen in humans. Indeed, the ob/ob animals in the present study hold alike dysfunction, evidenced by an impairment in HRV, demonstrated by decreased in either sympathetic and vagal cardiac modulation and total power, RMSSD, SD-PI, SD1, SD2, and alpha 1 as compared to controls. Importantly, only the female animals that underwent aerobic exercise training presented a subtle attenuation of HRV dysfunction. Once again, it seems that aerobic exercise training is better than resistance exercise training now in promoting positive effects on HRV. However, despite that some studies have demonstrated that an improvement on autonomic modulation leads to an improvement on cardiac function, we could not observe that association in this experimental model of obesity and type 2 diabetes.

Indeed, there are certain limitations of this study. We do not have a molecular or histological examination to confirm the physiological nature of hypertrophy. However, as discussed, obesity is associated with an increased LV mass. We also do not have a male group in this study. However, it is important to emphasize that about 80% of animal studies have been conducted in males, so, indeed, more studies using females are needed (Hughes, 2007; Clayton and Collins, 2014). Moreover, cardiovascular disease is the leading cause of death in women and two of the major cardiovascular risk factors are obesity and type 2 diabetes (Colditz et al., 1995; Barnes, 2011).

In conclusion, our results confirmed previous findings, showing that female ob/ob present hyperglycemia, glucose tolerance, reduction of HRV parameters and impairment in diastolic function. Additionally, all three types of exercise training attenuated body weight gain as well as reduced the glycaemia and glucose intolerance. However, only the animals underwent to aerobic exercise training showed an improvement in cardiac function and HRV. Most important, our results demonstrated that a better aerobic exercise capacity as well as a greater responsivity to aerobic exercise training make the effects of aerobic exercise training more evident. Thus, our data reinforce the role of aerobic exercise training, associated or not to resistance exercise training, as a non-pharmacologic approach to manage the cardiovascular risk by improving the cardiac autonomic modulation in women with overweight or obesity.

## Data Availability Statement

All datasets generated for this study are included in the article/supplementary material.

## Ethics Statement

All surgical procedures and protocols were approved by the ethics committee of the Universidade Nove de Julho (AN0021/2014) and were conducted in accordance with the Guide for the Care and Use of Laboratory Animals, issued by the National Institutes of Health.

## Author Contributions

FS-C contributed to interpretation of data, statistical analysis, and draft the manuscript. M-CI and BR contributed to conception and design of the work, interpretation of data, and draft the manuscript. MS contributed to conception and design of the work, acquisition of data, analysis and interpretation of data, statistical analysis, and draft the manuscript. AR contributed to acquisition of data. FS, JM, and DF contributed to acquisition of data, analysis, and interpretation of data. KD contributed to conception and design of the work, analysis and interpretation of data, statistical analysis, and draft the manuscript.

### Conflict of Interest

The authors declare that the research was conducted in the absence of any commercial or financial relationships that could be construed as a potential conflict of interest.
